# Brain structural and functional abnormalities in affective network are associated with anxious depression

**DOI:** 10.1186/s12888-024-05970-2

**Published:** 2024-07-25

**Authors:** Qiao Juan, Tao Shiwan, Sun Yurong, Shi Jiabo, Chen Yu, Tian Shui, Yao Zhijian, Lu Qing

**Affiliations:** 1 Department of Psychology, The Affiliated Xuzhou Eastern Hospital of Xuzhou Medical University, Xuzhou, 221004 China; 2 Department of Psychiatry, the Affiliated Brain Hospital of Nanjing Medical University, Nanjing, 210029 China; 3 School of Biological Sciences & Medical Engineering, Southeast University, Nanjing, 210096 China; 4 Child Development and Learning Science, Key Laboratory of Ministry of Education, Nanjing, China; 5 Nanjing Brain Hospital, Medical School of Nanjing University, Nanjing, 210093 China; 6 Department of Radiology, the First Affiliated Hospital of Nanjing Medical University, Nanjing, 210029 China; 7 West China Hospital, Mental Health Center, Sichuan University, Chengdu, 610047 China

**Keywords:** Gray matter volume, Functional connectivity, Anxious depression, Affective network

## Abstract

**Background:**

Anxious depression (AD) is a common subtype of major depressive disorder (MDD). Neuroimaging studies of AD have revealed inconsistent and heterogeneous brain alterations with the use of single-model methods. Therefore, it is necessary to explore the pathogenesis of AD using multi-model imaging analyses to obtain more homogeneous and robust results.

**Methods:**

One hundred and eighty-two patients with MDD and 64 matched healthy controls (HCs) were recruited. Voxel-based morphometry (VBM) was used to estimate the gray matter volume (GMV) of all subjects. The GMV differences between the AD and non-anxious depression (NAD) participants were used as regions of interest (ROIs) for subsequent resting state functional connectivity (rs-FC) analyses. Correlation analysis was used to evaluate the associations between clinical symptoms and abnormal function in specific brain areas.

**Results:**

Decreased GMV in the medial frontal gyrus (MFG) and the superior frontal gyrus (SFG) was observed in the AD group compared to the NAD group. Taking the MFG and SFG as ROIs, the rs-FC analysis revealed decreased FC between the left SFG and left temporal pole and between the left SFG and right MFG in the AD group compared to the NAD group. Finally, the FC between the left SFG and left temporal pole was negatively correlated with HAMD-17 scores in the AD group.

**Conclusion:**

By combining the GMV and rs-FC models, this study revealed that structural and functional disruption of the affective network may be an important pathophysiology underlying AD. The structural impairment may serve as the foundation of the functional impairment.

**Supplementary Information:**

The online version contains supplementary material available at 10.1186/s12888-024-05970-2.

## Introduction

Anxious depression (AD) is one of the most common subtypes of major depressive disorder (MDD), with a prevalence of 40–60% [1]. Compared to non-anxious depression (NAD), AD is more severe [2], has greater functional impairments [3, 4], a poorer prognosis [5], and a higher suicide rate [6]. These distinct clinical features and poorer prognosis suggest that different neurophysiological mechanisms may underlie AD as compared to NAD.

Using resting state magnetic resonance imaging (rs-MRI) to define the neurobiological subtypes of depression were made in the previous studies [7, 8]. So, it is a constructive strategy using the rs-MRI to explore the neurophysiological mechanisms of AD subtype. Neuroimaging studies of AD have revealed inconsistent and heterogeneous structural and functional alterations. Structural MRI studies have indicated that AD patients have structural brain alterations in the cortical-limbic circuit, which plays a role in emotion regulation [9, 10]. Further, thinning of the right medial orbitofrontal and fusiform gyri, the left temporal pole and the lateral occipital cortices has been observed in AD patients [11]. Additionally, decreased gray matter volumes (GMVs) in the insula and medial prefrontal cortices, which are known as the important areas of default mode and salience networks, have been observed in AD patients [12]. Functional MRI studies have indicated that the anterior insula, anterior cingulate, amygdala subregions and prefrontal cortex, which are strongly interconnected and form the key nodes of the affective and cognitive networks, display altered activity and functional connectivity (FC) in AD [13–17]. For instance, decreased FC between the amygdala subregions and the medial prefrontal cortex (mPFC) [18], increased FC in the posterior regions of the default mode network (DMN), and decreased FC in the anterior regions of the DMN have been observed [19]. A recent study reported decreased activity in the right orbital part of the middle frontal gyrus, and this was negatively correlated with retardation factor scores in AD patients [15].

In summary, patients with AD may exhibit characteristic structural and functional alterations in the affective network. However, the inconsistencies in the findings of studies using different models could be related to heterogeneity among the methods used. Of note, most studies to date have used a single model to investigate the neurophysiological mechanism or biomarkers of AD. Few studies have explored the intrinsic relationships between the structural changes and the characteristics of impaired FC in AD. In the present study, we combined two different models, voxel-based morphometry (VBM) and seed-based FC, to investigate the neurophysiological mechanism of AD. These combined models have been used in studies of the human brain and can offer a deeper understanding of structure-function relationships [18, 20].

Based on previous studies, the structural and functional brain alterations in AD are mainly present in the affective network, including the ventromedial prefrontal cortex (VMPFC), insula, amygdala, hippocampus, anterior cingulate and temporal pole [21]. Therefore, it was hypothesized that (1) compared with the NAD group, the AD group would exhibit alterations in GMV and related FC in the affective network, and structural abnormalities would be the basis of these functional abnormalities, and (2) the alterations in these brain functions in the affective network might be the neurophysiological mechanism of AD.

## Materials and methods

### Study design

This study performed a cross-sectional case-control study design. The demographic information and the MRI scan data were collected in MDD and healthy control (HC) groups, meanwhile the clinical scales were assessed in MDD patients.

### Participants

The sample comprised 98 subjects diagnosed with AD, 84 subjects diagnosed with NAD and 64 healthy controls (HC). All participants were recruited from the Affiliated Brain Hospital of Nanjing Medical University between September 2014 and December 2017. Patients with MDD were diagnosed based on the Diagnostic and Statistical Manual of Mental Disorders, fifth edition (DSM-V) and the Mini-International Neuropsychiatric Interview (MINI) [22]. The inclusion criteria for MDD patients were as follows: (1) between the ages of 18 and 55 years; (2) right-handed; (3) no mental disorders caused by organic diseases and/or psychoactive substances (e.g., hypertension, diabetes mellitus, thyroid disease); (4) no history of mental problems in the immediate family; and (5) no lactating or pregnant women. The severity of MDD was estimated by the Hamilton Depression Rating Scale (HAMD-17) [23] on the day of MRI scanning. Only patients who scored at least 17 were included in this study. The MDD patients were further divided into the AD group and NAD group based on their scores for the anxiety/somatic factor of the HAMD-17; that is, those with scores ≥ 7 were assigned to the AD group [9, 15, 24]. The HAMD-17 can be categorised into five symptomatic factors: cognitive disturbance, retardation, weight loss, sleep disturbance and anxiety/somatic factors [25]. Each factor comprises corresponding items [26].

HCs were recruited from the local community and matched to the patients based on age and gender. The MINI was administered to the HCs in order to exclude a history of psychiatric disorders. The HCs were all Han Chinese and right-handed. The inclusion criteria for the HCs were as follows: (1) subjects aged between 18 and 55 years; (2) no history of mental illness in first-degree relatives; (3) no somatic and/or neurological illnesses; (4) no substance abuse or dependence; (5) no pregnant or lactating women; (6) no MRI contraindications.

Each participant signed a formal informed consent form. The study was approved by the Ethics Committee of the Affiliated Brain Hospital of Nanjing Medical University.

### Imaging data acquisition

All subjects were scanned by a 3.0T MRI scanner (Siemens Trio, Erlangen, Germany). During scanning, all subjects were required to relax and stay still, but not fall asleep, and to avoid any systematic thinking. Head motion was minimized with the use of foam pads. High-resolution T1-weighted images were acquired with the following parameters: 176 axial slices; slice thickness = 1 mm; echo time (TE) = 2.48 ms; repetition time (TR) = 1900 ms; field of view (FOV) = 250 × 250 mm^2^; flip angle (FA) = 8°; matrix = 256 × 256. Image acquisition took 4 min 18 s. Bold images were acquired using gradient-recalled echo-planar imaging (GRE-EPI) with the following parameters: 32 slices; slice thickness = 4 mm; repetition time (TR) = 3000 ms; echo time (TE) = 40 ms; flip angle = 90°; field of view (FOV) = 240 × 240 mm^2^; matrix size = 64 × 64; voxel size = 3.75 mm× 3.75 mm. Image acquisition took 6 min 45 s.

### Preprocessing of the structure MRI data

Structural images were pre-processed with the VBM 8 toolbox (http://dbm.neuro.unijena.de/vbm) using SPM12 software (http://www.fil.ion.ucl.ac.uk/spm/software/spm12/) T1 images were normalized to a template space. Then, the images were segmented into gray matter (GM), white matter (WM) and cerebrospinal fluid (CSF). After preprocessing, modules were used to check the quality of the T1 images. Finally, the normalized images were smoothed with an 8-mm full-width by half maximum (FWHM) Gaussian kernel. After the above steps, abnormal structural images were excluded.

### Bold MRI data preprocessing

The preprocessing of the functional imaging data was conducted in SPM12 software (http://www.fil.ion.ucl.ac.uk/spm/software/spm12/) using MatlabR2013b as the platform. Before preprocessing, the first six volumes of time points were removed to eliminate the initial signal instability and noise from the machine. The remaining 127 volumes were preprocessed as follows: slice timing; spatial normalization to the standard Montreal Neurological Institute space with 3 mm isotropic voxels; linear detrending; temporal band-pass filtering (0.01–0.08 Hz); regression of nuisance signals, including Friston 24 head motion parameters, global signal, white matter signal and cerebrospinal fluid signal [27]. The resting data for all participants were excluded since head movements were > 2 mm and > 2° in any direction. After the above steps, seven subjects were excluded due to poor image quality, nine subjects were excluded due to a diagnosis of bipolar disorder, and 15 subjects, including two HCs, were excluded due to excessive head movement. Finally, 215 subjects, including 153 patients and 62 HCs, met the quality control criteria and were included in the analyses.

### Resting-state functional imaging analysis

Brain regions with significant differences in GMV between the AD/NAD groups were identified as masks. Then, the masks were set as regions of interest (ROIs). DPARSFA [28] was used to obtain the whole brain FC map of the ROIs. Next, a time series between the ROIs and other brain areas was extracted and averaged. Finally, Fisher’s r-to-z transform was used to convert the correlation coefficients to z values to improve the normality of the distribution.

### Statistical analysis

One-way analysis of variance (ANOVA) was used to compare the age, years of education and total HAMD-17 scores of the three groups. The chi-square test was used to compare categorical variables among the three groups. Independent sample t-tests were used to compare the HAMD-17 factors between the AD and NAD groups. *p*-values less than 0.05 were taken to indicate a statistically significant difference between the groups (two-tailed).

One-way analysis of covariance (ANCOVA) was used to calculate the differences in the GMV maps among the AD, NAD and HC groups. As the number of years of education differed among the three groups, it was entered as a covariate in the analysis. Although there were no significant differences in age and gender between the three groups, they were still included as covariates to eliminate their potential effects. Next, the clusters with significant GMV differences among the three groups were identified. Subsequently, post-hoc t-tests were used to calculate the GMV difference between each pair of groups based on the significant clusters from the ANCOVA analysis. Correction for multiple comparisons was performed using Gaussian random field correction (GRF correction). The cluster threshold was set at voxel *p* < 0.001, cluster *p* < 0.05, with a minimum cluster ≥ 283 voxels [29].

The clusters with significant GMV differences between the AD and NAD groups were chosen as the ROIs for the rs-FC analysis. A one-way ANCOVA was performed to evaluate the difference among the three groups, with age, education and gender as the covariates. Then, a voxel-based pair-to-pair comparison analysis was performed to identify the significant differences between each pair of groups, with the significantly different clusters among the three groups as the masks. The Alphasim program was used for correction for multiple comparisons. In these functional analyses, the threshold was set at voxel *p* < 0.001, cluster *p* < 0.05, and a minimum cluster ≥ 6 voxels.

After the above steps, Pearson’s partial correlation analyses were performed to examine the correlations between abnormal brain function and the HAMD-17 factors. Bonferroni correction was applied to adjust the alpha level. Because there were two functional connections and six HAMD-17 scores (HAMD-17 total score and five factor scores) entered into the correlation analysis. So, 12 multiple comparisons were made between Due to the number of multiple comparisons, the *p*-value was adjusted as follows: 0.05/12.

## Results

### Demographic and clinical features

There were no significant differences in gender and age between the three groups while the HC group had more years of education than the AD and NAD groups. The HAMD-17 score of the AD group was higher than that of the NAD group. There were no significant differences in the HAMD-17 factors between the AD group and the NAD group, except for the anxiety/somatic factor(See Table 1).

**Table 1:** The demographic and clinical characteristics of three groups

Measure(mean ± SD)	AD (*n* = 83)	NAD (*n* = 70)	HC (*n* = 62)	*p*-value
Age, year^a^	34.7 ± 10.45	31.67 ± 9.65	32.92 ± 9.87	0.17
Sex, male: female^b^	33/50	33/37	28/34	0.63
Education, year^a^	13.12 ± 3.17	14.06 ± 2.80	15.45 ± 2.76	0.00
HAMD score^c^	24.29 ± 4.22	19.42 ± 3.92	-	0.00
Anxiety/somatic ^c^	9.08 ± 1.65	4.46 ± 1.30	-	0.00*
Weight^c^	0.76 ± 0.85	0.64 ± 0.83	-	0.40
Retardation^c^	7.60 ± 1.33	7.27 ± 1.49	-	0.15
Cognition^c^	2.47 ± 1.80	2.01 ± 1.56	-	0.09
Sleep^c^	3.50 ± 1.88	3.50 ± 2.04	-	0.98

*Note* SD: standard deviation; AD: anxious depression; NAD: non anxious depression; HC: healthy control; HAMD: Hamilton Rating Scale for Depression. ^a^ indicates p values for one-way ANOVA; ^b^ indicates *p* values for chi-square test. ^c^ indicates *p* values for independent two-sample t-tests

### Differences in the GMV of the three groups

**Fig. 1:**
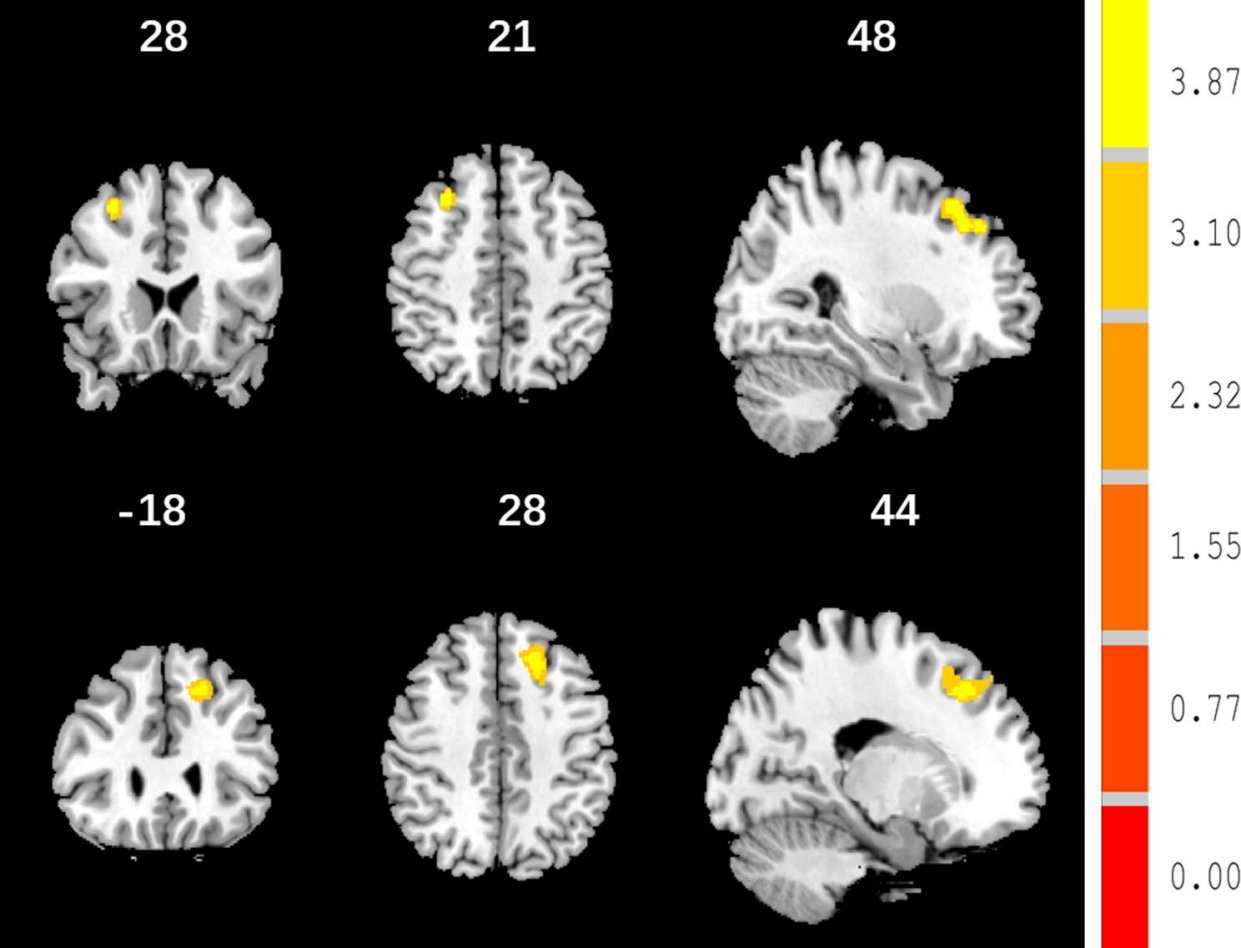
Differences in the GMV among the three groups. *Note* There were significant differences between the three groups in the right MFG and left SFG (*p* < 0.05, K > 283, GRF correction)

**Fig. 2:**
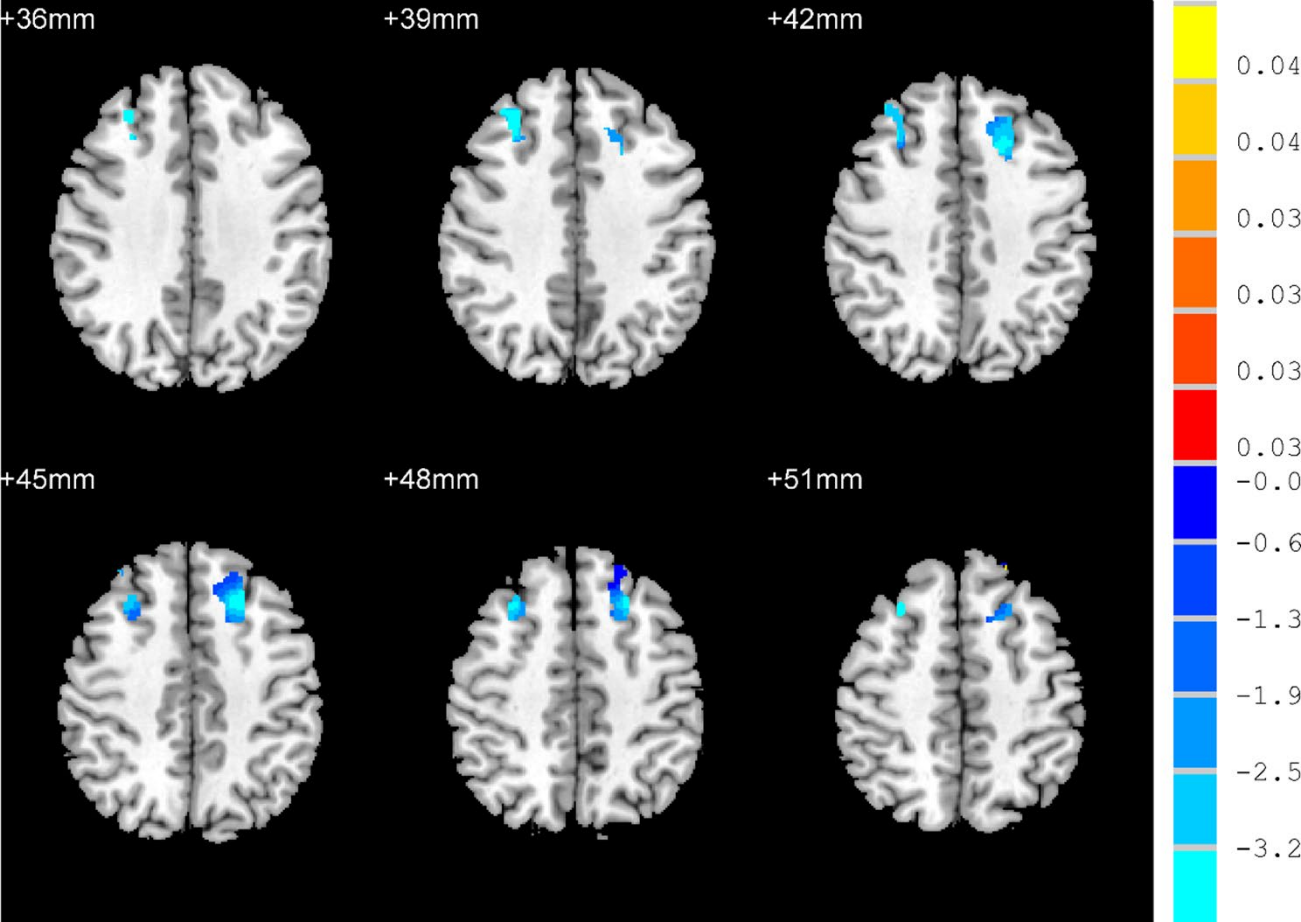
Differences in the GMV between the AD and NAD groups. *Note* Compared to the NAD group, the AD group showed reductions in the GMV of the SFG and MFG (colour bars indicated *t* values, *p* < 0.05, K > 283, GRF correction)

In the ANCOVA analysis, there were significant between-group differences in the GMVs of the right middle frontal gyrus (MFG) and left superior frontal gyrus (SFG) (See Table 2; Fig. 1) (GRF correction, *p* < 0.05). The AD group had a reduced GMV of the right MFG and left SFG relative to the NAD group. Compared to the HC group, the AD group exhibited a reduced GMV in the right MFG and left SFG while the NAD group showed an increased GMV. Therefore, the GMV in the right MFG and left SFG could be ordered as follows: NAD > HC > AD (Bonferroni correction, *p* < 0.05/3) (See Table 2; Fig. 2, Fig. S1 and Fig. S2).

**Table 2:** The difference of gray matter volume among three groups

Groups	Regions (AAL)	Peak point (MNI )			Cluster (voxels)	*F/t* (Peak point)
		X y z				
ANCOVA	R MFG	28.5	21	48	376	4.20*
	L SFG	-18	28.5	43.5	623	4.65*
AD < NAD	R MFG	28.5	21	48	376	-4.83
	L SFG	-18	27	43.5	623	-6.08
AD < HC	R MFG	30	28.5	39	376	-3.87
	L SFG	-22.5	25.5	45	623	-3.86
NAD > HC	L SFG	-19.5	37.5	48	623	3.31
	R MFG	28.5	16.5	43.5	373	2.04

*Note* AD, anxious depression; NAD, non-anxious depression; HC, healthy control; MNI, Montreal coordinate system; MFG: middle frontal gyrus; SFG: superior frontal gyrus; F, statistical value of the peak voxel showing the significant functional connectivity differences among all the groups; t, statistical value of the peak voxel showing significant functional connectivity differences between the AD and NAD, or NAD and HC. (*p* < 0.05, GRF correction, K > 283 voxels); * F value, others are t values

### Differences in FC between the three groups

**Fig. 3:**
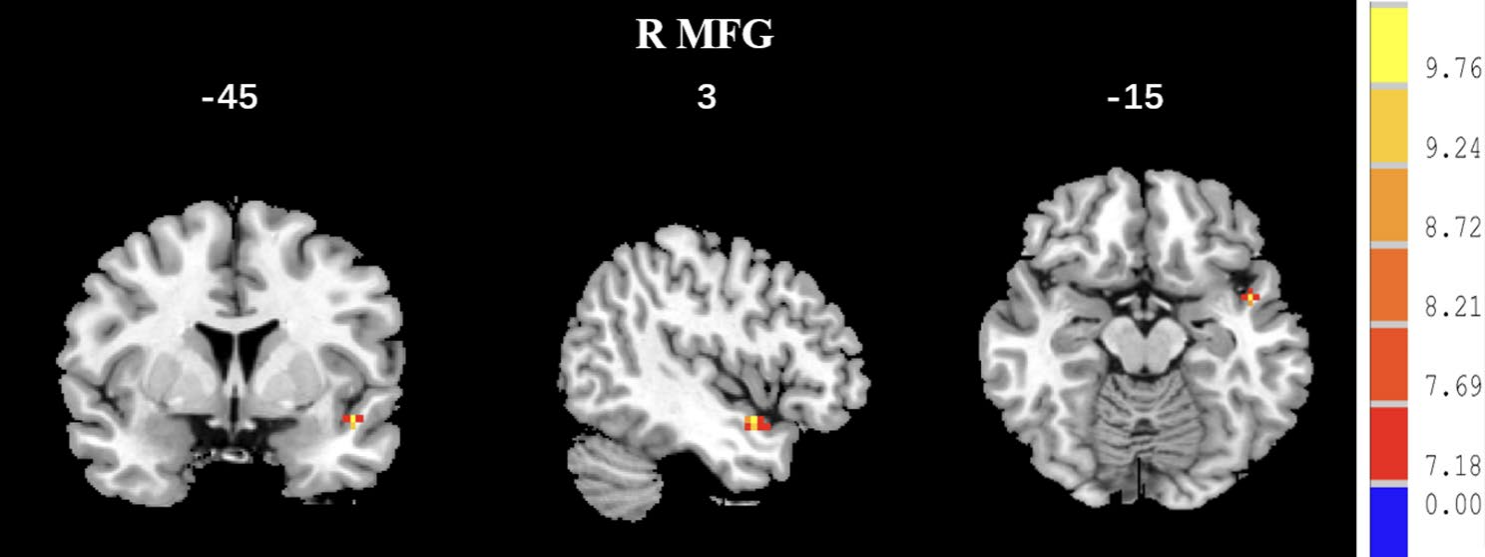
Differences in FC in the MFG among the three groups. *Note* With the right MFG as the ROI, there was a significant difference in the FC between the right MFG and left temporal pole (colour bars indicated *t* values, *p* < 0.05, K ≥ 6 voxels, Alphasim correction)

**Fig. 4:**
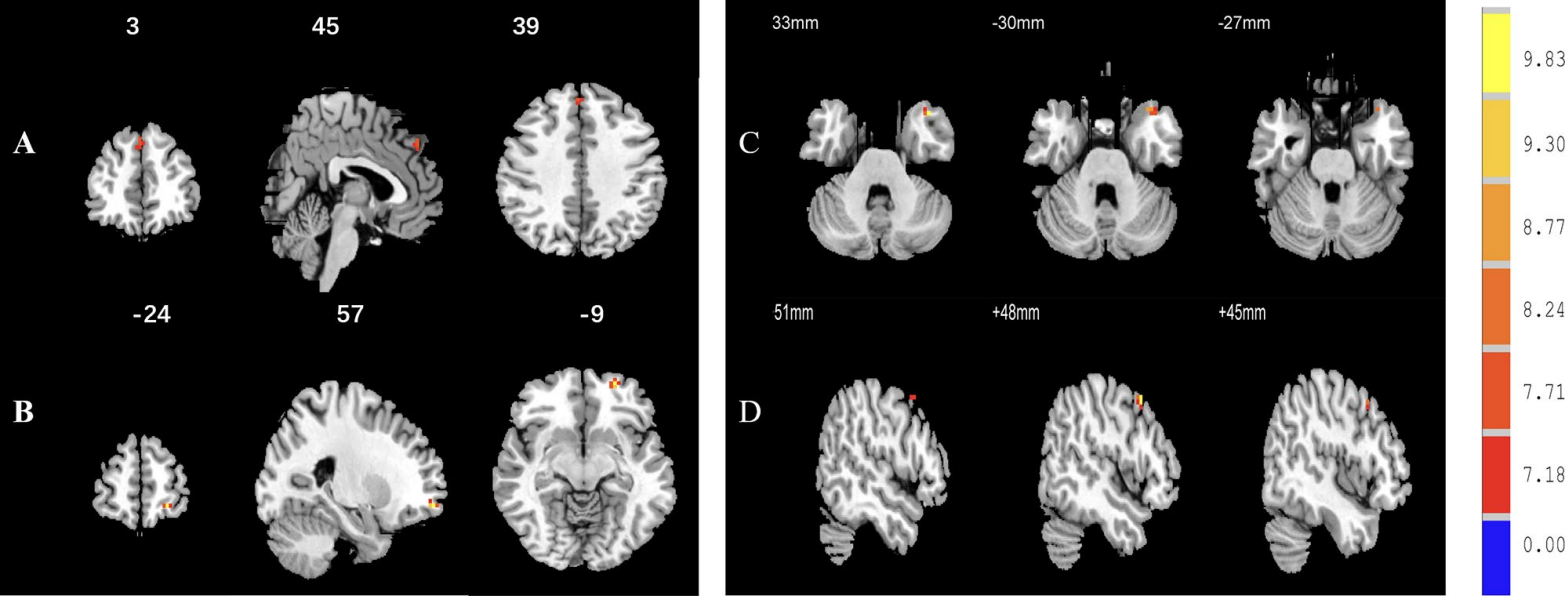
Differences in FC in the left SFG among the three groups. *Note* With the left SFG as the ROI, there were significant differences in FC between the SFG and the right superior frontal gyrus (see **A**), left middle frontal gyrus (see **B**), left temporal pole (see **C**) and right middle frontal gyrus (see **D**)

**Fig. 5:**
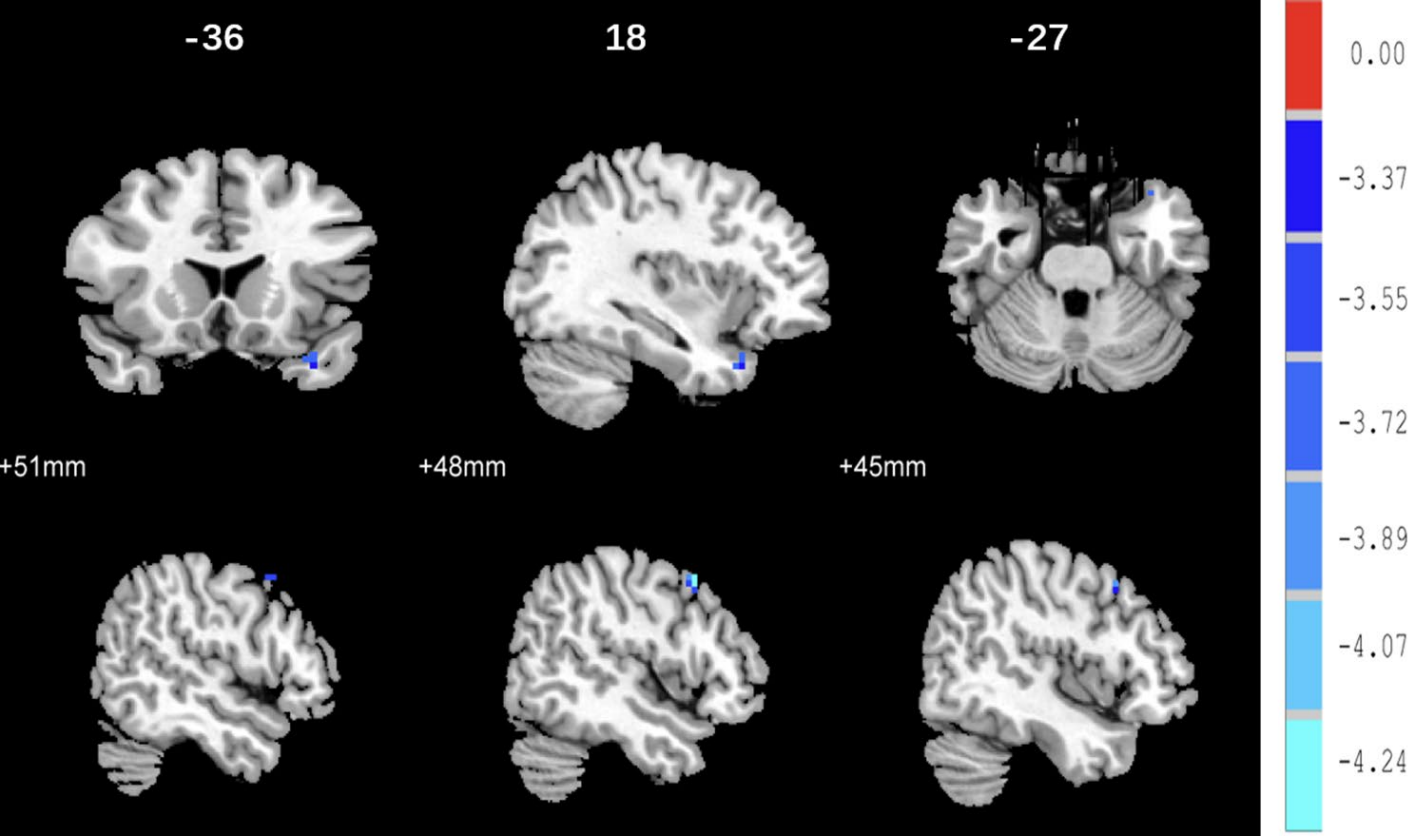
FC differences between the AD and NAD groups in the left SFG. *Note* (**A**) There was reduced FC between the left SFG and the left temporal pole in the AD group versus NAD group (*p* < 0.05, K ≥ 6 voxels, AlphaSim corrected). (**B**) The AD group showed decreased FC between the left SFG and the right middle frontal gyrus (*p* < 0.05, K ≥ 6 voxels, AlphaSim correction) as compared to the NAD group

With the right MFG and left SFG as the ROIs, ANCOVA analysis indicated that the FC between the right MFG and left temporal pole (See Fig. 3) and between the left SFG and the left middle temporal gyrus, left middle frontal gyrus, right superior frontal gyrus and right middle frontal gyrus was significantly difference among the three groups (AlphaSim corrected, *p* < 0.001, *p* < 0.05, k ≥ 6 voxels) (See Fig. 4). Compared to the NAD group, the FC between the left SFG and left temporal pole and between the left SFG and right middle frontal gyrus was significantly decreased in the AD group (AlphaSim corrected *p* < 0.001, *p* < 0.05, k ≥ 6 voxels) (See Fig. 5). Compared with the HC group, the AD group had reduced FC between the left SFG and left middle frontal gyrus (AlphaSim corrected, *p* < 0.001, *p* < 0.05, k ≥ 6 voxels) (See Fig. S3A). Compared with the HC group, the NAD group had increased FC between the left SFG and left temporal pole and between the right MFG and left temporal pole (AlphaSim corrected, *p* < 0.001, *p* < 0.05, k ≥ 6 voxels) (See Table 3, Fig. S3B and Fig. S4).

**Table 3:** The difference of the functional connectivity among three groups

	Region (AAL)	Peak Point			Cluster size	Peak	corrected *p*
		MNI				F/t	
ANOVA							
R MFG	L Temporal pole: superior	-45	3	-15	11	10.27	0.012
L SFG	L Middle temporal gyrus	-39	15	-33	10	10.33	0.023
	L Middle frontal gyrus, orbital part	-24	57	-9	9	10.37	0.035
	R Superior frontal gyrus, medial	3	45	39	7	7.74	0.045
	R Middle frontal gyrus	48	15	48	9	10.13	0.035
AD vs. NAD							
L SFG	L Temporal pole: middle	-36	18	-27	6	-3.79	0.037
	R Middle frontal gyrus	48	15	48	9	-4.41	0.002
AD vs. HC							
	L Middle frontal gyrus, orbital part	-24	57	-9	9	-4.44	0.002
NAD vs. HC							
L SFG	L Temporal pole: middle	-39	15	-33	7	4.17	0.028
R MFG	L Temporal pole: superior	-45	3	-15	11	4.24	< 0.001

*Note* MFG, Middle frontal gyrus; SFG, Superior frontal gyrus; MNI, Montreal Neurological Institute; AD, anxious depression; NAD, non-anxious depression; HC, healthy control; F, statistical value of the peak voxel showing the significant functional connectivity differences among all the groups; t, statistical value of the peak voxel showing significant functional connectivity differences between the AD and NAD, or NAD and HC. Corrected *p*, corrected by AlphaSim

### Correlations between the abnormal regions and clinical symptom in AD

**Fig. 6:**
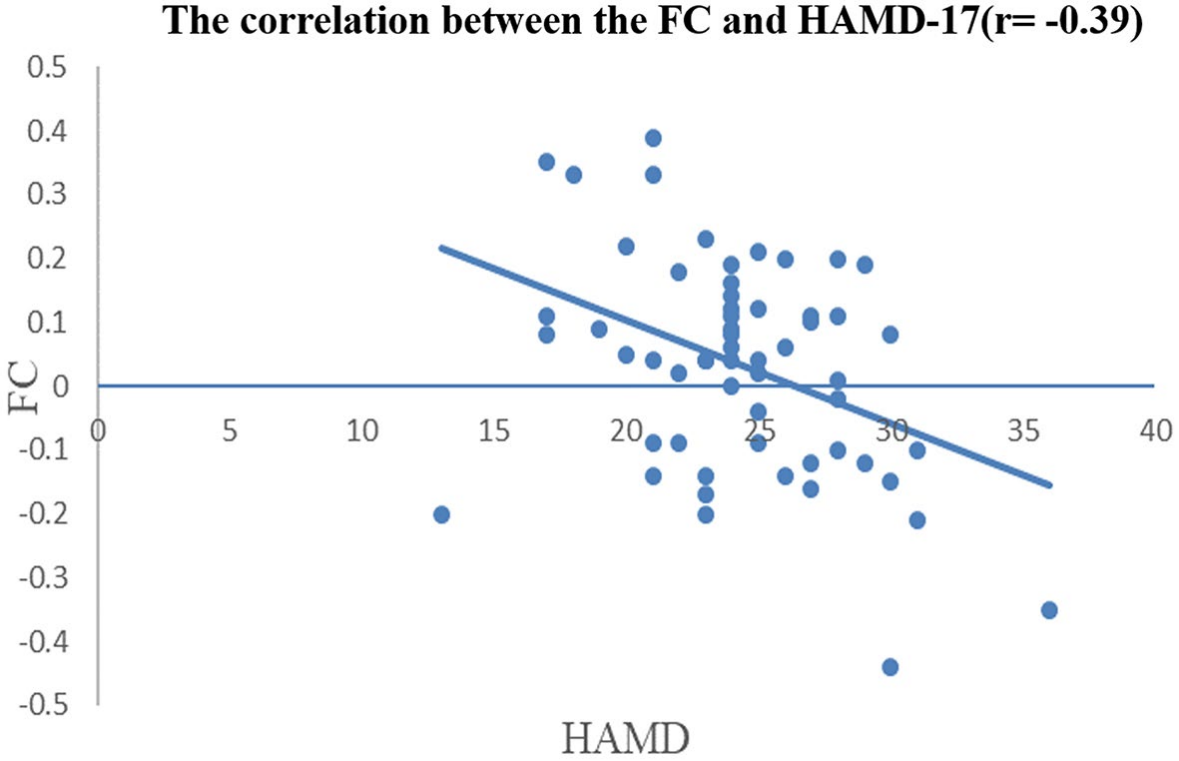
The negative correlation between the FC and the HAMD-17 in AD group. *Note* The X axis shows the HAMD-17 scores, while the FC between the left SFG and the left temporal pole in the AD group is presented on the Y axis (*p* < 0.05/12, Bonferroni corrected)

In the AD group, the FC between the left SFG and left temporal pole was negatively correlated with the HAMD-17. There were no other significant correlations between alterations in FC and clinical symptoms in the AD group. For the GMV, there were no significant correlations between the two abnormal regions and clinical features in the AD group. Subsequently, correlation analysis was performed between the two altered FCs and the total HAMD-17 score as well as the five HAMD-17 factors. A Bonferroni-corrected *p*-value of 0.05/12 was applied to these analyses. The results revealed that the FC between the left SFG and left temporal pole was negatively correlated with the HAMD-17 (*r* = -0.39, *p* = 0.002, Bonferroni corrected) (See Fig. 6).

## Discussion

In this research, structural and functional studies were combined to explore the neuropathology of AD. Firstly, the results revealed decreases in the GMVs of the MFG and SFG in the AD group relative to the NAD group. Interestingly, increased GMVs in these two regions were found in the NAD group relative to the HC group. Then, the differences in the GMVs between the AD and NAD groups were chosen as the ROIs to obtain a FC map of the whole brain. Differences in the FC between the right MFG and left temporal pole and between the left SFG and the left temporal pole, left MFG, right SFG and right MFG were observed among the three groups. Relative to the NAD group, decreased FC between the left SFG and the left temporal pole and right MFG was observed in the AD group. Compared to the HC group, decreased FC between the left SFG and left MFG was observed in the AD group. Increased FC between the left SFG and left temporal pole, and between the right MFG and left temporal pole, was observed in the AD group relative to the HC group. Finally, the FC and GMV were extracted to analyse the correlations between these metrics and clinical symptoms in the AD group. The results revealed that the FC between the left SFG and left temporal pole, which are the core nodes of the affective network, was negatively correlated with the HAMD-17 scores in the AD group. These findings suggest that an abnormality in the affective network may serve as the neuropathological basis of AD.

Our results are consistent with previous findings indicating that the prefrontal cortex (PFC) plays an important role in AD. In terms of the left SFG, our study showed a significant GMV reduction in AD patients relative to NAD patients. Prior studies have highlighted the critical role of the SFG in inhibitory control, self-awareness, cognitive control [30, 31] and emotion regulation-related processes [32]. These processes are hypothesized to play a role in increasing the vulnerability to pathological anxiety [33]. Further, the findings of the current study revealed a reduction in the GMV of the right MFG in AD group. The MFG is an important part of the dorsolateral prefrontal cortex (DLPFC), which is involved in working memory [34], cognitive control functions [35, 36] and attention [37, 38], especially in the top-down regulation of emotional processing [39]. Impairment of the DLPFC has been observed in nongeriatric depressed patients with anxiety [40], suggesting that dysfunctional top-down processing leads to state anxiety and depression.

In a previous study, compared to the NAD group, the AD group had larger GMVs than the MDD group in regions of the frontal and temporal lobes [41]. Peng and colleagues discovered a reduction in the GMV of the right inferior frontal gyrus and orbital frontal gyrus, which are involved in emotional regulation and sensory processing in anxious depression [9]. In another study, paediatric patients with anxious depression were found to have reduced GMVs in the DLPFC compared to paediatric patients with MDD alone [42]. Zhao et al. found that compared to NAD patients, AD patients had a reduced cortical thickness in the prefrontal, temporal and lingual lobes. Although the above studies present a heterogeneous and inconsistent picture of AD-specific neural alterations, the literature does highlight the important role of the DLPFC, which contains the MFG and SFG areas, in emotional regulation. Interestingly, in the current study, the NAD group had reduced GMVs in the MFG and SFG regions relative to the HC and AD groups. Many other studies have found an increased GMV in AD patients relative to NAD and HC groups. In adult patients, an increased brain volume may be related to neuronal differentiation, synaptogenesis, increased synaptic connections, and regional blood flow, thereby modifying the gray matter architecture [43]. In a clinical study, AD patients were found to have more severe symptoms [2], greater functional impairment [3, 4] and a poorer prognosis [5]. Based on its special clinical feature and distinct brain functional abnormality, it might be reasonable to conclude that AD may be characterised by much more abnormalities in brain function, which may be reflected by a reduction in the GMV. Under this theory, an increased GMV in NAD patients might reflect a compensatory increase in neuron volume. On the other hand, the reduced GMV in AD patients may be explained by a decompensation in neuron volume.

In addition, this study used the different GMVs between the AD and NAD groups to explore rs-FC differences between the AD vs. NAD and HC groups. Decreased FC was found between the left SFG and both the left temporal pole and right MFG in AD patients relative to NAD patients. The SFG and temporal pole belong to the affective network, which partially overlaps with the frontoparietal network (FPN). A previous study found significant correlations between anxiety symptoms and brain alterations in the FPN, indicating that impairments in the FPN may play a role in anxiety symptoms in late-life depression patients [40]. A study of general anxiety disorder (GAD) patients found reduced rs-FC of the positive connections in the STG and enhanced rs-FC of the negative connections in the inferior frontal gyrus, which is partially of the FPN [44]. A meta-analysis revealed disorder-specific GMV reductions in the fronto-limbic region in MDD and the fronto-temporal region in anxiety disorder [45]. Another transdiagnostic, multimodal meta-analysis of structural and functional MRI studies investigated the common and specific changes across MDD and anxiety disorders and reported that the disorder-specific changes included hyperconnectivity between the default and frontoparietal networks and hypoconnectivity between the limbic and salience networks in MDD while there was limbic network hyperconnectivity and reduced GMVs in the insular and medial-temporal cortices in anxiety disorders [12]. Further, the pure anxiety disorder group showed hypo- and hyperactivation in the temporal gyrus as well as hyperactivity in the SFG. Previous studies have demonstrated that the frontal and temporal regions are linked to higher cognitive involvement in anxiety disorders compared to MDD, which could explain the impaired fear processing [46].

In summary, the present study revealed decreased FC between the SFG and temporal gyrus in the AD group relative to the NAD group. This special FC abnormality in AD was similar to what is observed in anxiety symptom but nor depression symptom. Thus, it can be inferred that brain alterations in the affective network may be related to the anxious feature in MDD patients.

Finally, the correlation analysis in the current study indicated that the FC between the SFG and right temporal pole was negatively correlated with HAMD-17 in AD patients. The temporal pole cortex is generally referred to as the rostral end of the temporal lobe. It is highly connected to the DLPFC and parietal cortices [47] and is thought to play a role in audio-visual information integration [48], semantic memory, fluency and development [49], recognition of emotions and empathy [50]. GMV reductions have been reported in the PFC, frontal and temporal gyri, and temporal pole in a previous study [51]. The functional abnormalities in these brain regions may lead to negative cognitive bias, depressive contemplation, despair, social withdrawal, and even suicide in MDD patients [52]. In addition, based on the reduced GMV in the SFG, disruption of functional coordination in the SFG may be associated with structural abnormalities in this brain region.

There are several limitations of this study that should be noted. Firstly, there was a significant difference in the education level between patients and HCs, which may impact the results. Secondly, given that this was real-world research, it was difficult to restrict patients’ medication. Thus, the medication status and type of the patients may have affected the results of this study. In future studies, we aim to recruit drug naïve patients to eliminate the impact of medication and match the education level of the subjects to explore the neuropathological mechanisms of AD.

## Conclusion

The present study demonstrated specific structural and functional alterations in the affective network in AD patients. Reductions in the GMVs of the left SFG and right MFG were observed in AD patients. Further, decreased FC between the left SFG and the left temporal pole was observed in AD patients, and this was negatively correlated with HAMD-17 scores. Thus, it can be concluded that structural disruption may serve as the basis of FC deficits of the affective network in AD and functional deficits in the affective network could provide insights into the neurobiological basis.

### Electronic supplementary material

Below is the link to the electronic supplementary material.

Supplementary Material 1

Supplementary Material 2

Supplementary Material 3

Supplementary Material 4

Supplementary Material 5

## Data Availability

The data in this study is not publicly available due to ethical approval and confidentiality agreements made with participants, but are available from the corresponding author upon reasonable request.
