# Supplementary figures and images for: Brain structural and functional abnormalities in affective network are associated with anxious depression
Source: BMC Psychiatry. 2024 Jul 25;24:533. doi: 10.1186/s12888-024-05970-2 (PMC11270941; doi:10.1186/s12888-024-05970-2)

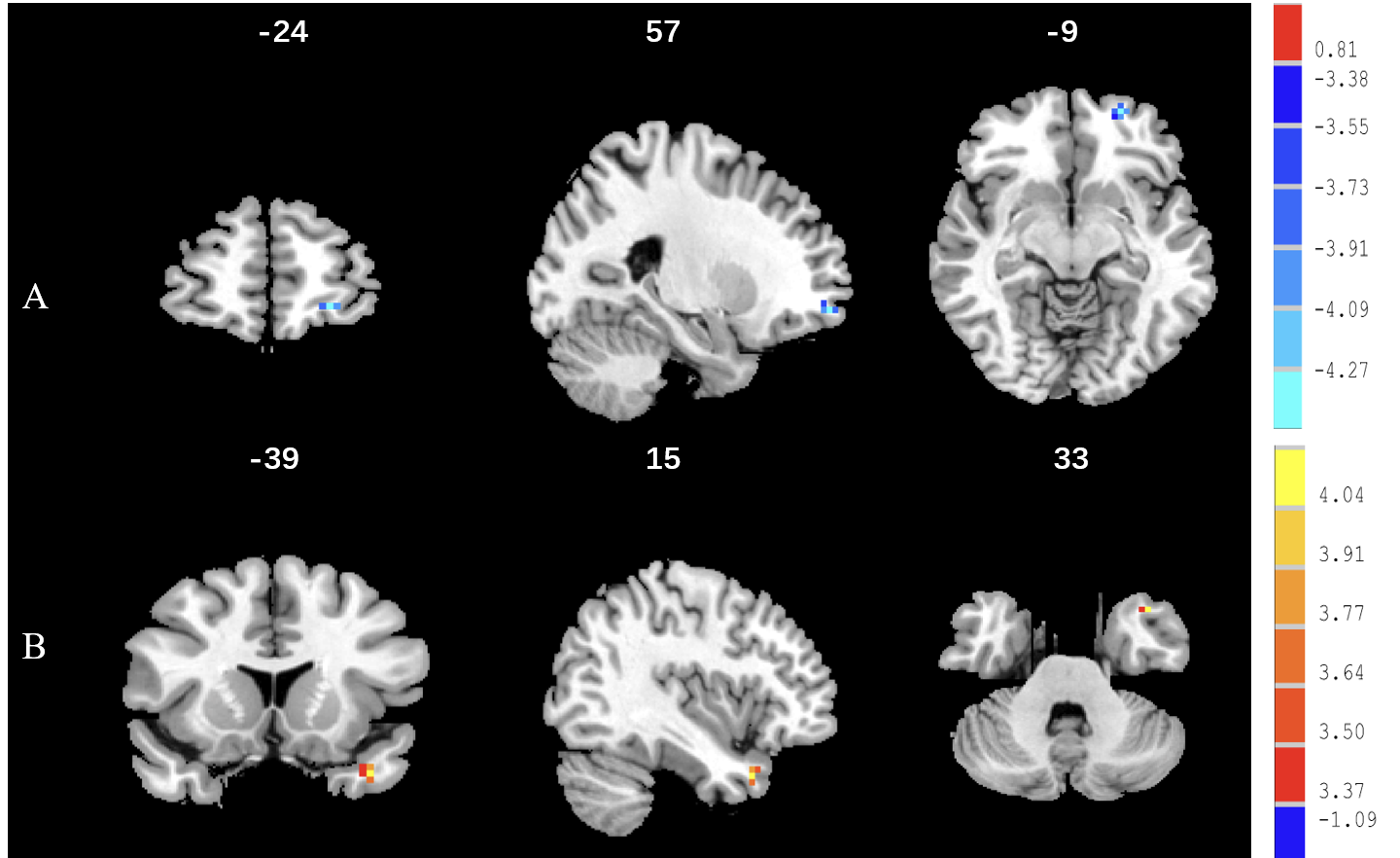

Supplement: Supplementary file 1 — Supplementary Material 1 [file 12888_2024_5970_MOESM1_ESM.tif]

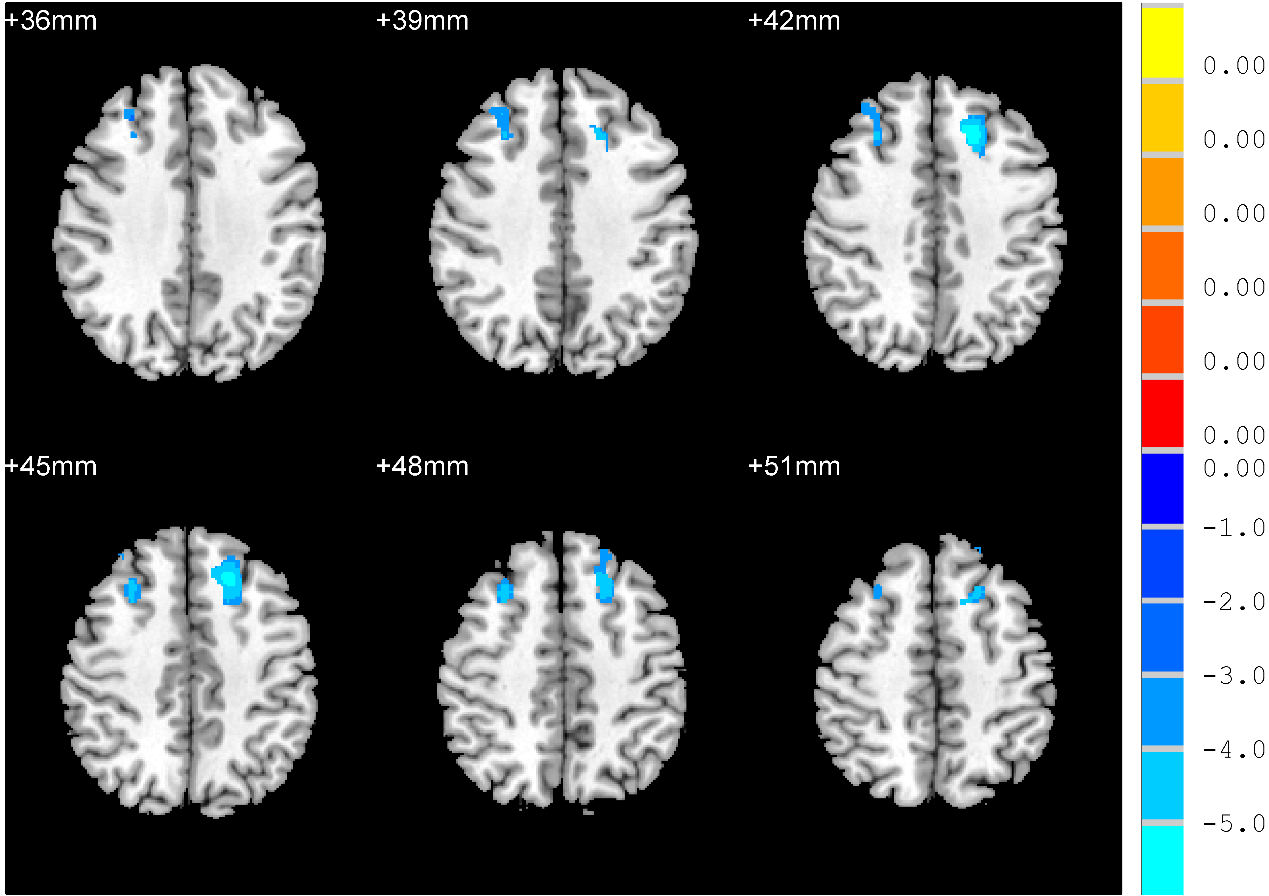

Supplement: Supplementary file 3 — Supplementary Material 3 [file 12888_2024_5970_MOESM3_ESM.tif]

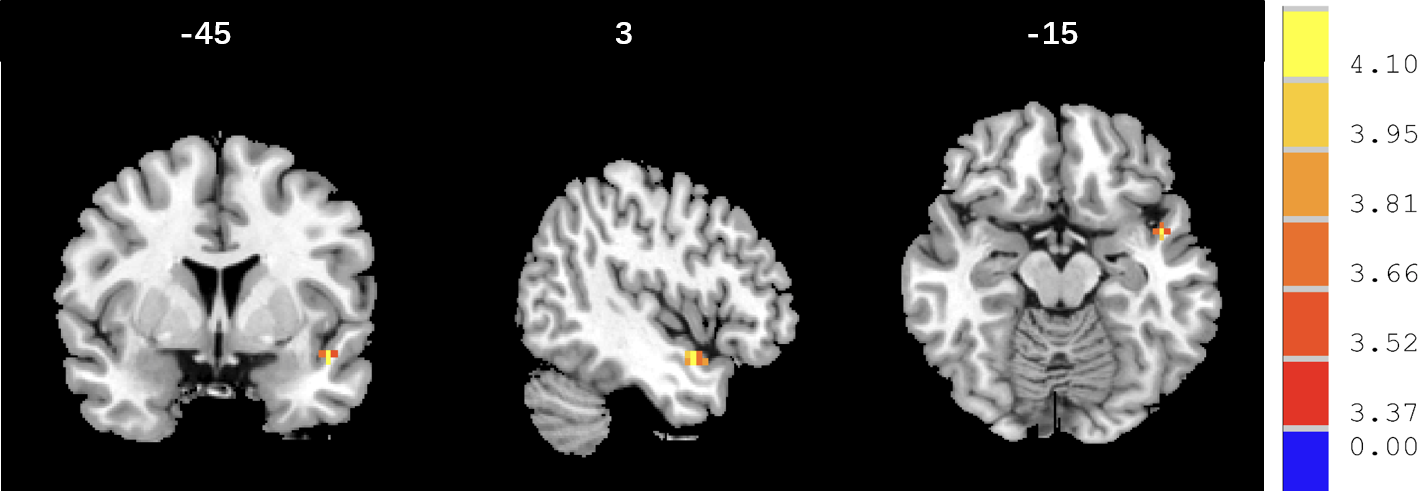

Supplement: Supplementary file 4 — Supplementary Material 4 [file 12888_2024_5970_MOESM4_ESM.tif]

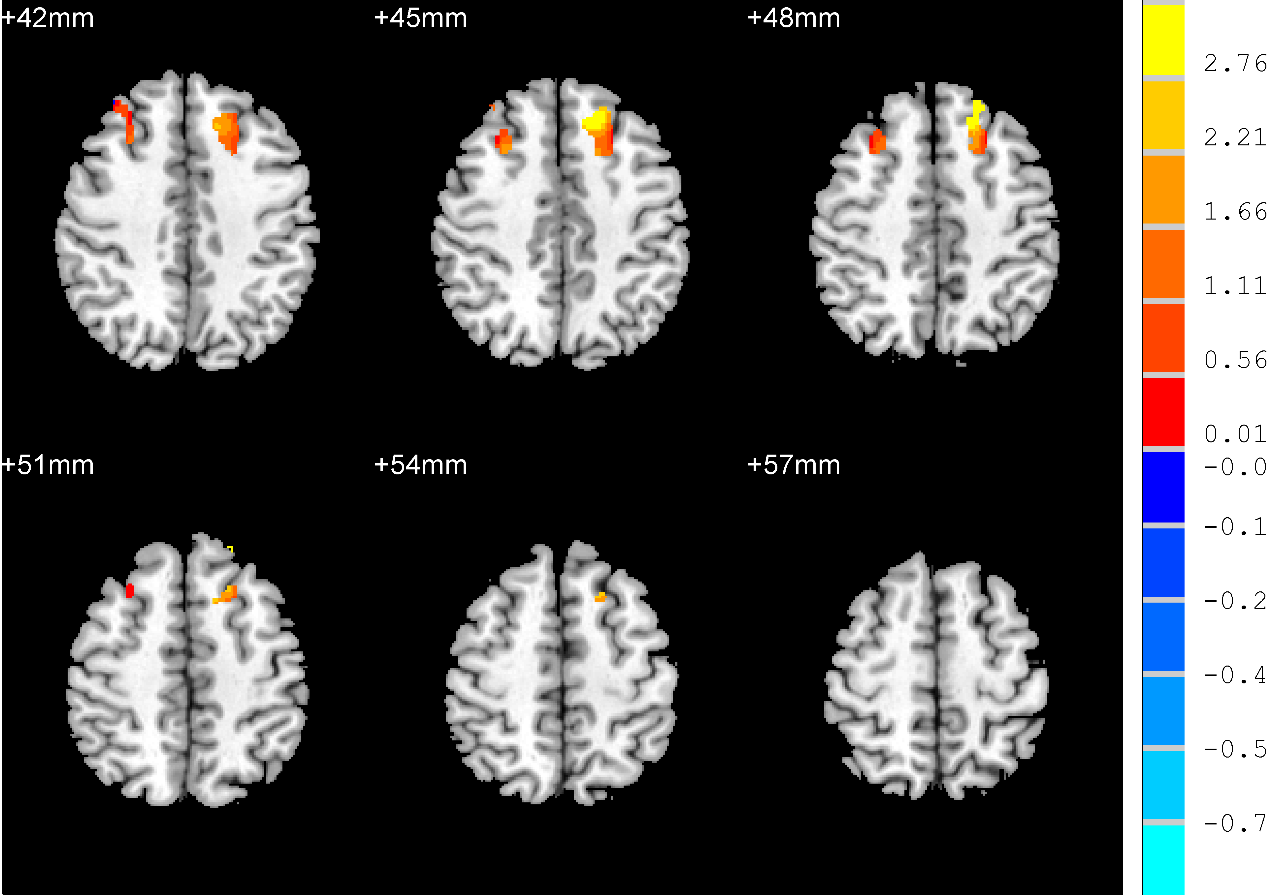

Supplement: Supplementary file 5 — Supplementary Material 5 [file 12888_2024_5970_MOESM5_ESM.tif]
