# Supplementary material for: Brain structural and functional abnormalities in affective network are associated with anxious depression
Source: BMC Psychiatry. 2024 Jul 25;24:533. doi: 10.1186/s12888-024-05970-2 (PMC11270941; doi:10.1186/s12888-024-05970-2)
Supplement: Supplementary file 2 — Supplementary Material 2 [file 12888_2024_5970_MOESM2_ESM.docx]

**Supplementary material**

**Figure S1** Differences of GMV between AD and HC group.

Note: Compared to HC group, AD showed decreased GMV in left SFG and right MFG. (color bar means *t* values, *p*＜0.05，K>283，GRF correction）

**Figure S2** Difference of GMV between NAD and HC group.

Note: Compared to HC group, NAD showed increased GMV in left SFG and right MFG. (color bar means *t* values, *p*＜0.05，K>283，GRF correction）

**Figure S3** The FC difference between AD/ NAD and HC group in left SFG.

Note: (A): Compared to HC group, AD showed decreased FC between left SFG and left middle frontal gyrus. (*p* < 0.05, K ≥ 6 voxels, AlphaSim correction) (B): Compared to HC group, NAD showed increased FC between left SFG and left temporal pole. (*p* < 0.05, K ≥ 6 voxels, AlphaSim correction)

**Figure S4** The FC difference between the NAD and HC group in right MFG.

Note: Compared to HC group, NAD showed increased FC between the right MFG and left temporal pole. (*p* < 0.05, K ≥ 6 voxels, AlphaSim correction)
